# Murine heart gene expression during acute Chagasic myocarditis

**DOI:** 10.1016/j.gdata.2015.03.009

**Published:** 2015-03-31

**Authors:** Andrés F. Henao-Martínez, Gabriel Parra-Henao

**Affiliations:** a Division of Infectious Diseases, Department of Medicine, University of Colorado Denver, United States; b Red Chagas Colombia, Instituto Nacional de Salud, Bogotá, Colombia and Grupo de Epidemiología y Bioestadística, Universidad CES, Colombia

**Keywords:** Chagas, Acute, Myocarditis, Murine, Gene expression

## Abstract

Chagas disease is transmitted by the parasite, *Trypanosoma cruzi*. Acute infection is characterized by acute myocarditis, although it is largely asymptomatic. Initial cardiac insult could be a determinant to the posterior development of chronic Chagasic cardiomyopathy, usually after 10 years in only approximately 30% of chronically infected patients. Herein, we characterized the acute gene expression profiling in heart tissue of two strains of mice infected with *T. cruzi* (tulahuen strain) at 4 weeks and their respective controls. Gene sequence data are available at NCBI under GEO accession number: GSE63847. The output of the genes expression suggests differences in involvement of protein kinase B (AKT), NCAM1, HLA-DRA, and ubiquitin C genes networks. These gene activation differences may correlate with myocardial contractility during the acute infection.

**Table:** 

Specifications	
Organism/cell line/tissue	Mus musculus
Sex	Male
Sequencer or array type	Agilent G3 SurePrint 8 + 60 Mouse Gene Expression One-Color Microarray Platform
Data format	Raw
Experimental factors	Infected Balb/c vs. infected C57BL/6J mice
Experimental features	7–8 weeks male Balb/c mice (N = 8) and C57BL/6J (N = 8) mice were infected with 150–200 tissue-derived trypomastigotes (*T. cruzi*; Tulahuen strain) and 8 Balb/c and 8 C57BL/6J were used as controls. 6 mice per group were used for gene expression analysis.
Consent	NA
Sample source location	Denver, Colorado, USA

## Direct link to deposited genomic data

http://www.ncbi.nlm.nih.gov/geo/query/acc.cgi?acc=GSE63847

## Experimental design, materials and methods

### Introduction

Chagas disease, one of the most neglected tropical diseases, has a complex pathophysiology [1]. Better understanding of this pathology may help in the clinical management of one of the planet's poorest affected population. This work aimed at contributing on the discovery of the host related mechanisms mediating the severity of acute Chagasic myocarditis, a possibly pivotal event in the development of chronic Chagasic cardiomyopathy [2], which is the main source of morbidity and mortality of this often fatal malady. Chagas disease is an infectious disease caused by the parasite *Trypanosoma cruzi*. Transmission occurs most frequently in endemic areas through the bite of an insect (Triatominae or kissing bugs). Initial Chagas infection does not produce many symptoms, however after an average of 10 years; around 30% of infected people develop heart disease [3]. The mechanisms responsible for the development of the delayed heart disease are not entirely known. Nevertheless, the injury produced in the heart during the initial infection might be an important contributor. Different strains of mice have different susceptibility to infection with *T. cruzi*. In this study, authors characterized myocytes injury based mainly on histopathology and echocardiograms early after Chagas infection in two strains of mice with different infection susceptibility. These findings were then correlated with genes expressed in their hearts. This data analysis was published recently [4]. This correlation showed that a network of genes, especially the AKT gene, could be associated with decreased heart muscle strength (contractility) during the initial infection [5]. This may be proven to be important to further identify potential host genes involved with an early worse cardiac response during the acute infection which could also be associated with the later development of chronic Chagas cardiac disease.

### Experimental design

7–8 weeks male Balb/c mice (N = 8) and C57BL/6J (N = 8) mice were infected with 150–200 tissue-derived trypomastigotes (*T. cruzi*; Tulahuen strain) and 8 Balb/c and 8 C57BL/6J were used as controls. 6 mice per group were used for gene expression analysis.

## Materials and methods

Mice were euthanized at 4 weeks post-infection. Hearts were harvested and the apex was cut and used for RNA extraction. Tissue was flash-frozen in liquid nitrogen, then disrupted, and homogenized using a TissueRuptor (Qiagen) followed by total RNA extraction using the RNeasy Plus Universal Mini Kit (Qiagen). RNA quality was determined using the Agilent 2100 Bioanalyzer with the RNA 6000 Nano Kit Assay. Gene expression profiling of heart tissue was performed on the Agilent G3 SurePrint 8 × 60 Mouse Gene Expression One-Color Microarray Platform. 6 mice per experimental group per strain were included for a total of 24 samples. Agilent's labeling kits for one-color assays were used followed by hybridization and wash procedures according to the manufacturer's protocols. Arrays were scanned on the Nimblegen MS200 High-Resolution Microarray Scanner on the 2-mm setting. Images were processed in the Agilent Feature Extraction software, and intensities were exported from the Feature Extraction software. Feature Extraction quality control (QC) reports were used to monitor data quality.

## Discussion

Host responses to acute injury may vary based on the underlying host genetic background [6]. These specific responses to organ injury might be in part responsible of different phenotypes during the acute infection and could correlate with chronic organ damage. The study evaluated differences in cardiac contractility among the two strains during acute infection [5]. Network of protein kinase B (AKT), NCAM1, HLA-DRA, and ubiquitin C genes are differently expressed among the two strains of mice during acute myocardial Chagas infection and may be responsible for the contractility differences seen among the mice strains. Further studies are necessary to modify or manipulate the genes expression. This will help to characterize their role in the acute cardiac response to infection and the development of the chronic cardiomyopathy. Additionally human studies of chronic Chagasic cardiomyopathy may be prove useful evaluating the prognostic capacity of these molecules as potential biomarkers. Other genes are also differentially expressed and could also be potentially studied [5].
